# Severe Lung Dysfunction and Pulmonary Blood Flow during Extracorporeal Membrane Oxygenation

**DOI:** 10.3390/jcm13041113

**Published:** 2024-02-16

**Authors:** Lars Falk, Marika Lidegran, Sandra Diaz Ruiz, Jan Hultman, Lars Mikael Broman

**Affiliations:** 1 ECMO Centre Karolinska, ME Pediatric Perioperative Medicine and Intensive Care, Karolinska University Hospital, Akademiska Straket 14, 171 76 Stockholm, Sweden; jan.hultman45@gmail.com (J.H.); lars.broman@regionstockholm.se (L.M.B.); 2 Department of Physiology and Pharmacology, Karolinska Institutet, 171 76 Stockholm, Sweden; 3 Department of Pediatric Radiology, Astrid Lindgren Children’s Hospital, Karolinska University Hospital, 171 76 Stockholm, Sweden; marika.gullberg-lidegran@regionstockholm.se (M.L.); sandra.diaz.ruiz@ki.se (S.D.R.); 4 Department of Women’s and Children’s Health, Karolinska Institutet, 171 76 Stockholm, Sweden; 5 Department of Radiology, Lund University, 221 00 Lund, Sweden

**Keywords:** extracorporeal membrane oxygenation, septic shock, sepsis, pulmonary blood flow, prolonged ECMO, prognostication, prognosis, tidal volume

## Abstract

Background: Extracorporeal membrane oxygenation (ECMO) is indicated for patients with severe respiratory and/or circulatory failure. The standard technique to visualize the extent of pulmonary damage during ECMO is computed tomography (CT). Purpose: This single-center, retrospective study investigated whether pulmonary blood flow (PBF) measured with echocardiography can assist in assessing the extent of pulmonary damage and whether echocardiography and CT findings are associated with patient outcomes. Methods: All patients (>15 years) commenced on ECMO between 2011 and 2017 with septic shock of pulmonary origin and a treatment time >28 days were screened. Of 277 eligible patients, 9 were identified where both CT and echocardiography had been consecutively performed. Results: CT failed to indicate any differences in viable lung parenchyma within or between survivors and non-survivors at any time during ECMO treatment. Upon initiation of ECMO, the survivors (*n* = 5) and non-survivors (*n* = 4) had similar PBF. During a full course of ECMO support, survivors showed no change in PBF (3.8 ± 2.1 at ECMO start vs. 7.9 ± 4.3 L/min, *p* = 0.12), whereas non-survivors significantly deteriorated in PBF from 3.5 ± 1.0 to 1.0 ± 1.1 L/min (*p* = 0.029). Tidal volumes were significantly lower over time among the non-survivors, *p* = 0.047. Conclusions: In prolonged ECMO for pulmonary septic shock, CT was not found to be effective for the evaluation of pulmonary viability or recovery. This hypothesis-generating investigation supports echocardiography as a tool to predict pulmonary recovery via the assessment of PBF at the early to later stages of ECMO support.

## 1. Introduction

Extracorporeal membrane oxygenation (ECMO) has been used for decades in neonates and children with severe refractory cardiac and/or respiratory problems [1]. After the H1N1 epidemic in 2009, ECMO also became the mainstay treatment for the most severe adult respiratory cases [2]. ECMO is a technology to support patients with refractory severe acute respiratory distress syndrome (ARDS) and offers several benefits where conventional intensive care does not suffice. ECMO patients can usually be awake, communicate, and perform physiotherapy [3,4]. As more complex cases have been treated with ECMO over the years, treatment time has increased. The definition of prolonged ECMO has been pushed forward and is now considered to be >28 days of support [5], mostly to reflect COVID-19-related ARDS [6]. Increased acceptance of and experiences with longer treatment times come with the downside of awake patients risking a “bridge to nowhere”, i.e., futile cases where the patient’s lung function does not recover [7]. It is important to realize that ECMO is not a destination therapy but primarily indicated for potentially reversible organ failure or as a bridge to decision. However, ECMO has been successfully used as a bridge to lung transplantation in patients with end-stage respiratory failure or to heart transplantation or the implantation of ventricular assist devices [7]. The use of ECMO in new patient groups, e.g., septic shock [8], and improvements in technology have allowed more patients a chance for recovery, albeit sometimes necessitating prolonged support [9].

Assessment of respiratory function according to Extracorporeal Life Support Organization (ELSO) guidelines is usually performed before trial-off and weaning, which includes a thorough evaluation of blood gases and ventilatory settings [10]. However, these assessments often include information from other investigations such as computed tomography (CT) [11]. Today, CT is often used to evaluate lung recruitability and lung pathology [12,13]. A conventional CT scan can assist in the assessment of the amount of aerated lung but can have difficulties visualizing details of a consolidated lung. However, a contrast-enhanced CT scan is a valuable tool to visualize perfused lung parenchyma [14,15].

Furthermore, echocardiography is essential for daily evaluations of cardiac function, pulmonary blood flow (PBF), etc. From these measurements, other physiological variables, e.g., pulmonary vascular resistance (PVR), can be derived [16,17,18]. PVR, for example, increases in pathologies such as thromboembolism, lung fibrosis, and other parenchymal disorders [19]. Furthermore, an elevated baseline PVR was associated with higher 60-day mortality in ARDS patients offered conventional intensive care [20]. Additionally, in a prospective observational study on 226 patients with moderate to severe ARDS, 22% expressed right ventricular dysfunction, which was identified as an independent predictor of 28-day mortality, carrying a 60% mortality rate [21]. In ECMO patients with ARDS secondary to bacterial pneumonia, mortality has been reported to be similar or higher at 67% [22].

This work aims to investigate whether there is any association between the extent of pulmonary injury assessed using CT and physiological data on PBF obtained by means of echocardiography and how these factors relate to patient outcomes in terms of mortality.

## 2. Materials and Methods

**Figure 1:**
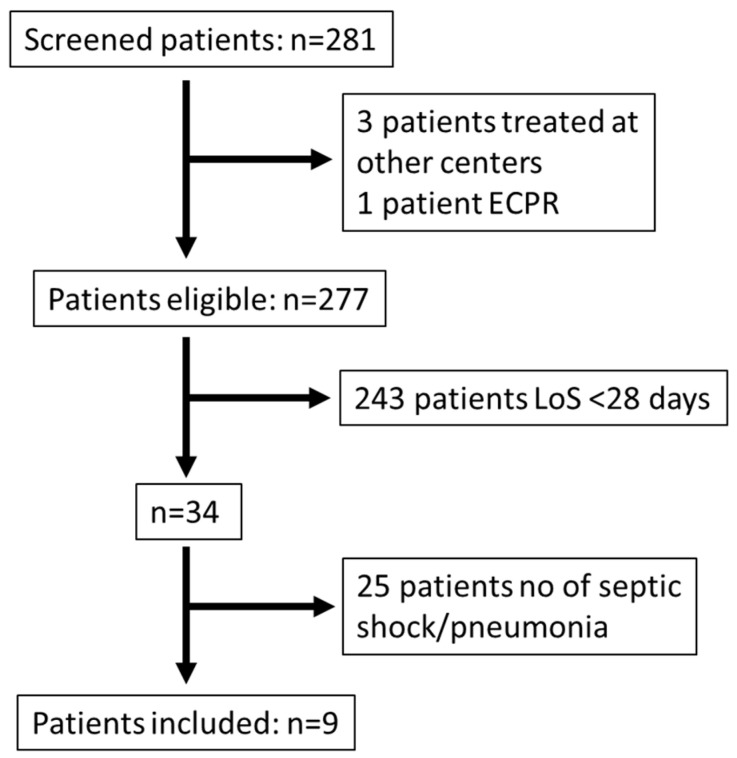
Patient selection. Inclusion: 15 years of age or older, ECMO support at ECMO Centre Karolinska between 2011 and 2017 with septic shock according to Sepsis 2, originating from pneumonia, and treated for >28 days. Patients were excluded if they received extracorporeal cardiopulmonary resuscitation or were partly treated with ECMO at another hospital. Abbreviations: ECPR, extracorporeal cardiopulmonary resuscitation; LoS, length of stay.

All patients, 15 years of age or older, offered ECMO support at our unit between 2011 and 2017 were screened. Patients with septic shock according to Sepsis 2 [23] (given the study time period), originating from pneumonia, and treated for >28 days were eligible for inclusion. The exclusion criteria were treatment at another center during part of the ECMO support or cannulation for extracorporeal cardiopulmonary resuscitation (Figure 1).

### 2.1. Data Collection

Data were retrospectively collected from the hospital’s medical charts. The demographic data included age, body weight, height, sex, Sequential Organ Failure Assessment (SOFA) score [24], Survival After VenoArterial ECMO (SAVE) score [25], Respiratory Extracorporeal Membrane Oxygenation Survival Prediction (RESP) score [26], and Simplified Acute Physiology Score 3 (SAPS3) [27]. Treatment time for ECMO was divided into five time points. T0 at admission, T25 at 25% of the unique patient’s time on ECMO, and then T50, T75, and T100, at 50%, 75%, and 100% of the patient’s support time, respectively. At these pre-determined time points, the following variables were recorded together with echocardiography and CT examinations: ECMO mode, sedation (Richmond Agitation–Sedation Score [28]), spontaneous or controlled breathing, tidal volume, fraction of viable lung parenchyma, PBF, ECMO blood flow, right ventricular pressure, and PVR. If data were not accessible at the end of ECMO (T100), the last recorded data were used. For all other sampling points, data or examinations closest to the pre-set date (±5 days) were recorded. These five time points were used to describe the trajectory of organ dysfunction during ECMO treatment.

### 2.2. Computed Tomography Examinations

All CT examinations were performed on a second-generation dual-source CT scanner (Definition Flash; Siemens^®^ Medical Systems, Forchheim, Germany). Standard protocols for the examined body region were used. Radiation doses were kept to a minimum using automated tube voltage selection, automatic tube current modulation, and iterative reconstruction. Standard contrast doses were used: 2 mL/kg (maximum dose 120 mL) Visipaque at 320 mg I/mL. A previously published protocol for contrast administration adapted to the patient’s specific ECMO circulation was followed (12).

The CT examinations were retrospectively evaluated on a PACS workstation (Sectra PACS, Sectra AB, Linköping, Sweden) by three senior radiologists well experienced in CT and other imaging techniques in ECMO patients. Qualitative and pseudo-quantitative assessment of the images was independently performed and in the case of discrepancies, a final result was reached by consensus. Viable lung parenchyma was defined as an aerated lung or condensed but perfused parenchyma, measuring >40 HU (Hounsfield units) on contrast-enhanced CT scans. The visual estimation of the percent of viable lung parenchyma was related to 100% of the total lung on each side.

### 2.3. Echocardiography

The exams were obtained from hospital charts. Only exams where the velocity time integral (VTI) was calculated over the proximal part of the main pulmonary artery (MPA) were included. PBF was calculated according to Equation (1) [29]:

PBF = HR × (DMPA2 × π /4) × VTIMPA(1)

where HR is the heart rate and D is the diameter of the MPA. Data on admission (designated T0) as well as additional laboratory data, vasoactive inotropic score (VIS) [30], and vasopressor need were collected at T0, T25, and T100.

### 2.4. Statistical Analysis

Data were controlled for normality using Shapiro–Wilk’s test. Categorical data are presented as numbers (n) and fractions (%), normally distributed data are presented as means (±1 SD), and non-parametric data are presented as medians (IQR 25–75%). Analysis of parametric data was performed using a *t*-test while non-parametric data were compared using the Mann–Whitney U-test. In cases with <5 observations in one group, a t-test was used. For comparisons of categorical data, Fisher’s exact test or the Chi^2 test were used. A mixed effects model was used to analyze repeated measurements over time. A *p*-value < 0.05 was considered a significant difference.

## 3. Results

Nine patients were included in the study (67% females) with an age of 33.1 ± 13.9 years (range 15–59). All patients were assessed, cannulated, and retrieved on ECMO from other hospitals in Sweden and northern Europe by our mobile ECMO service. The median mechanical ventilation time at the start of ECMO was 2 days (range 1–14 days). The mean VIS was 43 ± 37. Additional data at admission are available in Table 1. Seven patients were initiated on venoarterial (VA) ECMO, one was initiated on veno-venoarterial (VVA), and one was initiated on venovenous (VV) ECMO (Table 2). From here on, VVA mode will be included in the VA group.

### Basic Data of Survivors and Non-Survivors

There were no differences between survivors and non-survivors concerning age, days from intubation to ECMO, or length of ECMO treatment. However, at admission, the SAPS3 score was lower in survivors (56 ± 9) compared to the non-survivors (82 ± 12, *p* = 0.006).

Over the course of treatment, the number of patients on VA ECMO reduced in favor of VV support; VA decreased from 89% at T0 to 55% at T100. At the last point, 80% of the survivors were supported with VV ECMO whilst all non-survivors were on VA ECMO (*p* = 0.048) (Table 2). Survivors were significantly more awake according to their Richmond Agitation–Sedation Score (RAAS) at T100 (*p* = 0.00). Among the non-survivors, there was no difference in awakeness at T0 compared to T100 (*p* = 0.13).

**Figure 2:**
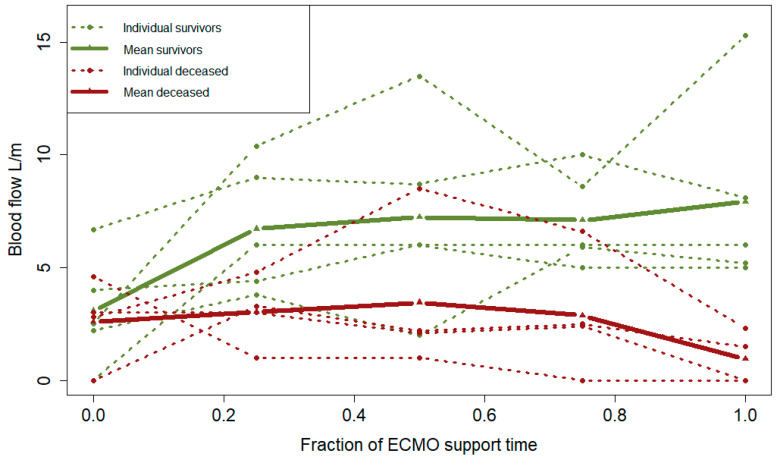
Interaction between survivors and non-survivors (mixed effects model) regarding pulmonary blood flow over time for the individual patients (dotted lines) and aggregated for the respective group (filled lines) (*p* = 0.00). The red color marks non-survivors, and the green color marks survivors. Abbreviations: ECMO, extracorporeal membrane oxygenation.

**Figure 3:**
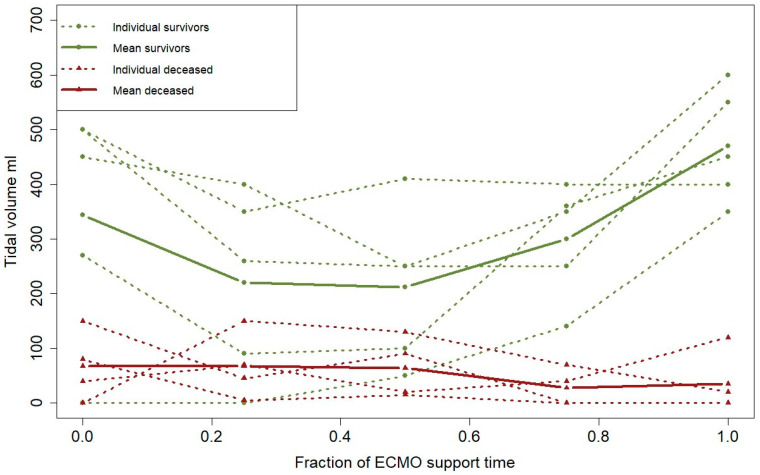
Interaction over time between survivors and non-survivors (mixed effects model) of tidal volume (mL) for the individual patients; red dotted lines indicate non-survivors and green dotted lines indicate survivors. Aggregated data are displayed for the respective group with filled lines (*p* = 0.047). Abbreviations: ECMO, extracorporeal membrane oxygenation.

**Figure 4:**
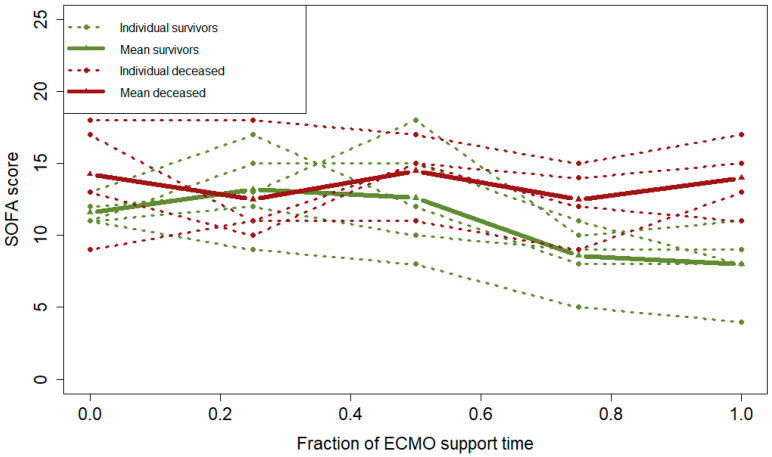
Interaction over time between survivors and non-survivors (mixed effects model) of SOFA for the individual patients (dotted lines) and aggregated for the respective group (bold lines) (*p* = 0.03). The red color marks non-survivors, and the green color marks survivors. Abbreviations: SOFA, Sequential Organ Failure Assessment score; ECMO, extracorporeal membrane oxygenation.

There were inconsistencies in the clinical picture, i.e., tidal volumes and the CT exams pertaining to respiratory function (Table 3 and Table 4). Tidal volumes were significantly lower among the non-survivors, but at the same time, the CT scans did not demonstrate any significant difference in estimated viable lung parenchyma. PBF indicated a trend of improvement between T0 and T100 for the survivors (*p* = 0.08). However, there was no trend of improvement observed among the non-survivors (*p* = 0.31). Furthermore, there was a significant difference in the trajectory slopes of PBF between survivors and the deceased (*p* = 0.004), indicating that the survivors maintained or increased PBF over the course of treatment (Figure 2). Additionally, a difference between survivors and non-survivors regarding tidal volumes (*p* = 0.047) and SOFA score (*p* = 0.03) indicated continued deterioration in lung function, or failing lung recovery, as well as aggravated multiorgan failure for the non-survivors (Figure 3 and Figure 4). Right ventricular pressure did not differ over the course of treatment or between T0 and T100 between survivors and non-survivors (Table 3 and Table 4).

Five patients survived (56%) to hospital discharge and were still alive at 5-year follow-up. Four patients were offered bilateral lung transplantations of whom two survived and were still alive at 5-year follow-up. Two patients died in connection with the transplantation from generalized bleeding at the sites of anastomosis between the recipient and the new organ. The survivors from lung transplantation had preserved tidal volumes and PBF compared to the non-survivors from lung transplantation.

## 4. Discussion

In this retrospective single-center study, we investigated the association between findings from CT scans and echocardiography with the degree of lung damage in a subset of patients on prolonged ECMO with bacterial pneumonia, ARDS, and septic shock. Our findings show that persistently low non-recovering tidal volumes and decreased PBF may indicate a poor outcome. The results showed that initial PBF was low and remained low over time in non-survivors, whereas BPF was higher and tended to increase over time in survivors. Furthermore, tidal volumes were preserved and increased over the course of ECMO support in survivors, contrary to the non-survivors where the tidal volumes remained low without signs of recovery. CT scan or assessment of right ventricular pressure was not able to predict outcomes in this setting.

The findings from this study illustrate the difficulties in assessing the prognosis of patients in need of ECMO for a prolonged time. Usually, assessment is performed through daily clinical evaluation of lung and circulatory recovery via bedside examinations, CT, and other imaging techniques. However, an examination does not always corroborate the actual state of the disease as exemplified by the weak discrimination CT scans offered for this particular population. However, CT may be of great benefit in patients with interstitial disease to determine the extent of parenchymal involvement [31]. On the other hand, the variable value of CT to determine lung viability in conventionally treated ARDS patients has been reported [32,33,34,35]. Patients can be treated with ECMO despite complexities such as extensive consolidations, pleural effusions, pneumothorax, and hemothorax, even in cases of severe necrotizing bacterial pneumonia. Given the multifactorial layout of a CT scan, the difficulties in evaluating the extent of pulmonary damage become apparent. These changes may lead to both over- and underestimation of the consolidated areas due to, e.g., compression of alveoli or emphysematous parts.

The role of echocardiography to evaluate the physiology of the lung may be of great value in these patients and contribute to the assessment of the prognosis as well as an indirect sign of the extent of pulmonary damage. Even at T25, PBF was significantly reduced in non-survivors compared to survivors. Likewise, survivors showed comparably higher preservation of tidal volume at commencement and during the course of ECMO support. There are several studies that highlight the importance of pulmonary vascular resistance in ARDS patients. Lammi et al. reported an association between increased baseline-indexed pulmonary vascular resistance and 60-day mortality in non-survivors [36]. In another prospective trial on 226 patients with moderate to severe ARDS, right ventricular dysfunction was identified as an independent predictor of 28-day mortality [21].

With prolonged ECMO, new questions arise, such as whether correct care is provided and if ECMO may be a bridge to recovery. These questions may be more difficult to answer compared to conventional ECMO patients, since the lungs may be more damaged by necrosis or parenchymal destruction. A longer time on ECMO also raises ethical questions for the clinician. Should the ECMO support be withdrawn or should we continue [7]? In this deliberation, the last remaining question will be whether the patient is eligible for lung transplantation, an issue actualized by the recent COVID-19 pandemic [37].

Five of our nine patients survived, a number comparable to ECMO data in general, as reported by the ELSO Registry [38]. The deceased exhibited limited tidal volumes and decreasing pulmonary blood flows over the treatment period. Four patients underwent lung transplantation following ECMO. The non-survivors from transplantation died from uncontrollable intrathoracic bleeding. Ongoing inflammatory processes could have contributed to this bleeding. Inflammation may also explain why these patients experienced continuous respiratory deterioration over the course of ECMO support. One may deliberate on whether these patients could have been treated differently in the earlier stages of disease. For example, it has been suggested that pneumectomy could be performed at an early stage to minimize inflammation with surrounding tissue damage and perform lung transplantation when the inflammation has subsided [39,40]. Several studies have shown acceptable results in ECMO patients with ARDS bridged to lung transplantation [37,41,42,43]. However, in these studies, the initial insult often took place weeks before the transplantation and the extent of inflammation at the time of surgery may have therefore been negligible. As such, it is difficult to extrapolate these results to case studies where pneumectomy preceded later transplantation [39,40]. On the contrary, other data support the benefits of early lung transplantation within the first two weeks of ECMO [44]. In the aforementioned study, Oh and colleagues showed a significant difference in survival between short-term bridge and long-term bridge to transplant on ECMO, where patients receiving ECMO for more than 14 days were exposed to a three times higher risk of death at 1-year follow-up [44].

The current results, although based on only a few patients, hypothesize that the evolution of PBF, tidal volume, and SOFA score may be important contributors to prognostication in prolonged ECMO due to pulmonary septic shock. Furthermore, an improvement in PBF, increased tidal volumes, and a reduction in SOFA score may indicate a favorable prognosis. However, an absence of improvement or deterioration in these variables may prompt a discussion about eligibility for lung transplantation. The timing of transplantation is difficult. An early decision may benefit the patient in terms of outcome [44]. However, transplantation is also a path of no return, and surviving with native lungs may be better for long-term survival. To venture on to speculation, in patients where experience states that lung transplantation is the only way forward, maintained PBF and tidal volume may indicate a favorable outcome. As to the contrary, diminished PBF, continued loss of tidal volume, and a persistently high SOFA score indicates poor outcomes for these very sick patients.

The main future implication of this study is not to dismiss CT as a useful modality but to recognize the limitations of the naked human eye and conventional software tools available to the radiologist. Note, this study was resourced with three experienced radiologists interpreting the CT examinations. Evaluation of machine learning algorithms, i.e., the use of artificial intelligence, deserves to be investigated and considered in this setting [45,46]. The value of ultrasound technology in the hands of the intensivist is further established.

### Limitations

This study was limited by the inherent problems of all retrospective studies. The rather small study sample makes this study hypothesis-generating. The generalizability of results originating from a single high-volume ECMO center is also limited. Furthermore, due to the problem of retrospectivity, the researchers struggled with how to divide and assort the data on a case level and agreed on the division of each treatment time into the five time slots. This measure is related to the individual’s time on ECMO support, which carries the limitation that case development cannot be foreseen. Moreover, in lung transplant patients, the individual’s time on the waitlist is dependent on how long it takes to find a suitable donor. Additionally, there is a limitation related to the subjective evaluation and scoring of CT images, although this was mitigated by having several examiners.

## 5. Conclusions

Computed tomography angiography, which is widely used to assess pulmonary viability and recovery, was not supportive of prognostication in this population of ECMO patients on prolonged treatment for septic shock and pneumonia. Echocardiography was useful in evaluating trends for PBF, which, together with tidal volume and SOFA score, separated the survivors and the deceased over time. Prospective studies are needed.

## Figures and Tables

**Table 1:** Abbreviations: ALT, alanine aminotransferase; AST, aspartate aminotransferase; BUN, blood urea nitrogen; p-lactate, plasma lactate; BMI, body mass index; FiO_2_, fraction of inspired oxygen; P/F, ratio of arterial oxygen partial pressure to fractional inspired oxygen; SOFA, Sequential Organ Failure Assessment score; SAVE, Survival After VenoArterial ECMO score; RESP, Respiratory Extracorporeal Membrane Oxygenation Survival Prediction score; SAPS3, Simplified Acute Physiology Score 3; paO_2_, arterial oxygen partial pressure; paCO_2_, arterial carbon dioxide partial pressure; VV, venovenous extracorporeal membrane oxygenation; VA, venoarterial extracorporeal membrane oxygenation; ECMO, extracorporeal membrane oxygenation. Normally distributed data are presented as the mean (±1 SD), and non-parametric data are presented as the median (IQR25%–IQR75%). Numbers may be specified, and fractions from the whole group are shown in (%).

Data on Admission for Extracorporeal Membrane Oxygenation (N = 9)	
Age, years	33.1 ± 13.9 (range: 15–59)
Weight, kg	77 ± 21.1
Length, cm	168 ± 9.9
BMI	27 ± 4.9
Male sex, %	33
FiO_2_	1.0 (range: 1.0–1.0)
P/F ratio, mmHg	50.7 ± 16.1
paO_2_, kPa [mmHg]	6.3 ± 3 [47 ± 22]
paCO_2_, kPa [mmHg]	8.3 ± 3 [62 ± 22]
Days with mechanical invasive ventilation	2(1–8.8)
Mean arterial blood pressure, mmHg	69 ± 14
p-Lactate, mmol/L	3.40(1.5–4.4)
Cardiac arrest prior to ECMO, *n*(%)	1 (11)
Vasoactive inotropic score	43 ± 37 (range: 2–116)
SOFA score	12.8 ± 2.9 (range: 9–18)
SAPS 3 score	67 ± 17 (range: 43–95)
SAVE score (VA)	−6 ± 5.2 (range: −15–−2)
RESP score (VV)	2.3 ± 4.7 (range: −3–6)
Transport on ECMO, ground, *n*(%)	3 (33)
Aircraft, *n*(%)	6 (67)
Cardiac diagnosis, *n*(%)	3 (33)
Cardiac arrest before ECMO, *n*(%)	1 (11)
Acute kidney injury, *n*(%)	6 (67)
Chronic renal failure, *n*(%)	0 (0)
Liver failure (acute), *n*(%)	3 (33)

**Table 2:** Abbreviations: VV, venovenous; V-VA, veno-venoarterial; VA, venoarterial; †, deceased; L-Tx, lung transplanted from extracorporeal membrane oxygenation.

Modes During Extracorporeal Support						
No.	T0	T25	T50	T75	T100	Outcome
1	V-VA	V-VA	V-VA	V-VA	VV	Survived
2	VA	VA	VA	VA	VA	†; L-Tx, cerebral bleeding one week after transplant
3	VA	VV	VV	VV	VV	Survived, L-Tx (and subsequent renal Tx)
4	VA	VA	VA	VA	VA	†; L-Tx, died from intrathoracic bleeding in the 1st post-op
5	VA	VA	VA	VA	VA	Survived, L-Tx
6	VV	VV	VV	VA	VA	†;
7	VA	VV	VV	VV	VV	Survived
8	VA	VA	VA	VA	VA	†; ECMO withdrawn due to futility
9	VA	VA	VA	VA	VV	Survived

Pink background color signifying VA ECMO and blue background color signifying VV ECMO.

**Table 3:** Comparison between survivors and the deceased, respectively, between T0 and T100. * Insufficient data. Abbreviations: SOFA, Sequential Organ Failure Assessment score.

	*p*-Value T0 vs. T100 (Paired *t*-Test)	
Variable	Survivors (*n* = 5)	Non-Survivors (*n* = 4)
Tidal volume (mL)	0.16	0.57
Viable lung parenchyma (%)	0.69	*
Right ventricular pressure (mmHg)	0.55	0.48
Pulmonary blood flow (L/min)	0.08	0.31
SOFA	0.03	0.87

**Table 4:** Mixed effects model comparing survivors and non-survivors over time. Abbreviations: SOFA, Sequential Organ Failure Assessment score.

Variable	*p*-Value Interaction Time and Survivors vs. Non-Survivors (Mixed Effects Model)
Tidal volume (mL)	0.047
Viable lung parenchyma (%)	0.85
Right ventricular pressure (mmHg)	0.10
Pulmonary blood flow (L/min)	0.00
SOFA	0.03

## Data Availability

All data generated or analyzed during this study are included in this published article. Original data may be provided upon reasonable request.
